# Oxaliplatin Eluting CalliSpheres Microspheres for the Treatment of Unresectable or Recurrent Hepatocellular Carcinoma

**DOI:** 10.3389/fphar.2022.923585

**Published:** 2022-08-11

**Authors:** Yonghua Bi, Kewei Ren, Jianzhuang Ren, Ji Ma, Xinwei Han

**Affiliations:** Department of Interventional Radiology, The First Affiliated Hospital of Zhengzhou University, Zhengzhou, China

**Keywords:** hepatocellular carcinoma (HCC), drug-eluting beads transarterial chemoembolization (DEB-TACE), CalliSpheres beads, oxaliplatin, TACE

## Abstract

**Aim:** Drug-eluting beads-transarterial chemoembolization (DEB-TACE) has been widely used in unresectable and advanced hepatocellular carcinoma (HCC). However, no study reported the clinical outcomes of drug-eluting beads TACE (DEB-TACE) with oxaliplatin-eluting CalliSpheres microspheres in the treatment of HCC. This study reports the preliminary outcomes of DEB-TACE loaded with oxaliplatin for the treatment of patients with unresectable or recurrent HCC.

**Methods**: From November 2019 to November 2021, 29 patients with unresectable or recurrent HCC were recruited from our department and treated by DEB-TACE loaded with oxaliplatin. The primary endpoint was progression-free survival (PFS), and the secondary endpoints were disease control rate and safety. Tumor response was investigated at 1, 3, and 6 months after DEB-TACE according to the criteria of the response evaluation in solid tumor (RECIST) criteria and the modified RECIST criteria (mRECIST). Survival curve was generated with the Kaplan–Meier method.

**Results:** A total of 49 DEB-TACE sessions were performed, with a technical success rate of 100%. The overall response rate and disease control rate were 52.4 and 95.2%, 64.7 and 76.5%, and 54.5 and 63.3%, respectively, at 1, 3, and 6 months after DEB-TACE (mRECIST). The PFS was 5.9 months, and the median overall survival was 18.8 months. The 6- and 12-month overall survival rate was 82.5% and 67.5%, respectively, No treatment-related mortality or severe adverse events were observed. Minor complications were observed in 21 patients (72.4%), and abdominal pain (41.4%) was the most common treatment-related complication.

**Conclusion:** DEB-TACE loaded with oxaliplatin-eluting CalliSpheres microspheres could be a safe, feasible, and efficacious palliative regimen in unresectable or recurrent HCC patients.

## Introduction

Hepatocellular carcinoma (HCC) is one of the most common malignant tumors and the leading cause of cancer-related mortality worldwide (Torre et al., 2012; Mittal and El-Serag, 2013). Unfortunately, more than 70% of patients are diagnosed with late-stage HCC and cannot be treated with curative treatments such as transplantation or liver resection (Mazzaferro et al., 2014; Morise et al., 2014), so the prognosis of HCC remains poor. Transarterial chemoembolization (TACE), as an efficacious palliative treatment, has been widely used in patients with unresectable and advanced HCC (Llovet and Bruix, 2003; Bruix and Sherman, 2011). Oxaliplatin is the third generation of platinum anticancer drugs and shows an excellent inhibitory effect on various cancers (Ibrahim et al., 2004; Nobili et al., 2008). However, adverse effects, such as cardiotoxicity and neurotoxicity, are observed in some patients due to high blood concentration (Bullinger et al., 2011).

Drug-eluting beads TACE (DEB-TACE), as a relatively new drug-delivering device, showed an advantage of sustained release of chemotherapy drugs and increased local drug concentration (Wu et al., 2018; Bi et al., 2021a). DEB-TACE with oxaliplatin-eluting hepasphere microspheres has been used to reduce toxicities for patients with unresectable intrahepatic cholangiocarcinoma and liver metastasis of colorectal cancer (Poggi et al., 2008; Poggi et al., 2009). CalliSpheres microsphere is the first drug-eluting bead developed in China, which has been applied for loading and releasing oxaliplatin in an *in vitro* study (Han et al., 2019). However, no study reported the outcomes of oxaliplatin-eluting CalliSpheres microspheres in the treatment of HCC. In this study, we aim to report the preliminary outcomes of DEB-TACE loaded with oxaliplatin for the treatment of patients with unresectable or recurrent HCC.

### Patients and Methods

#### Selection of Patients

**FIGURE 1:**
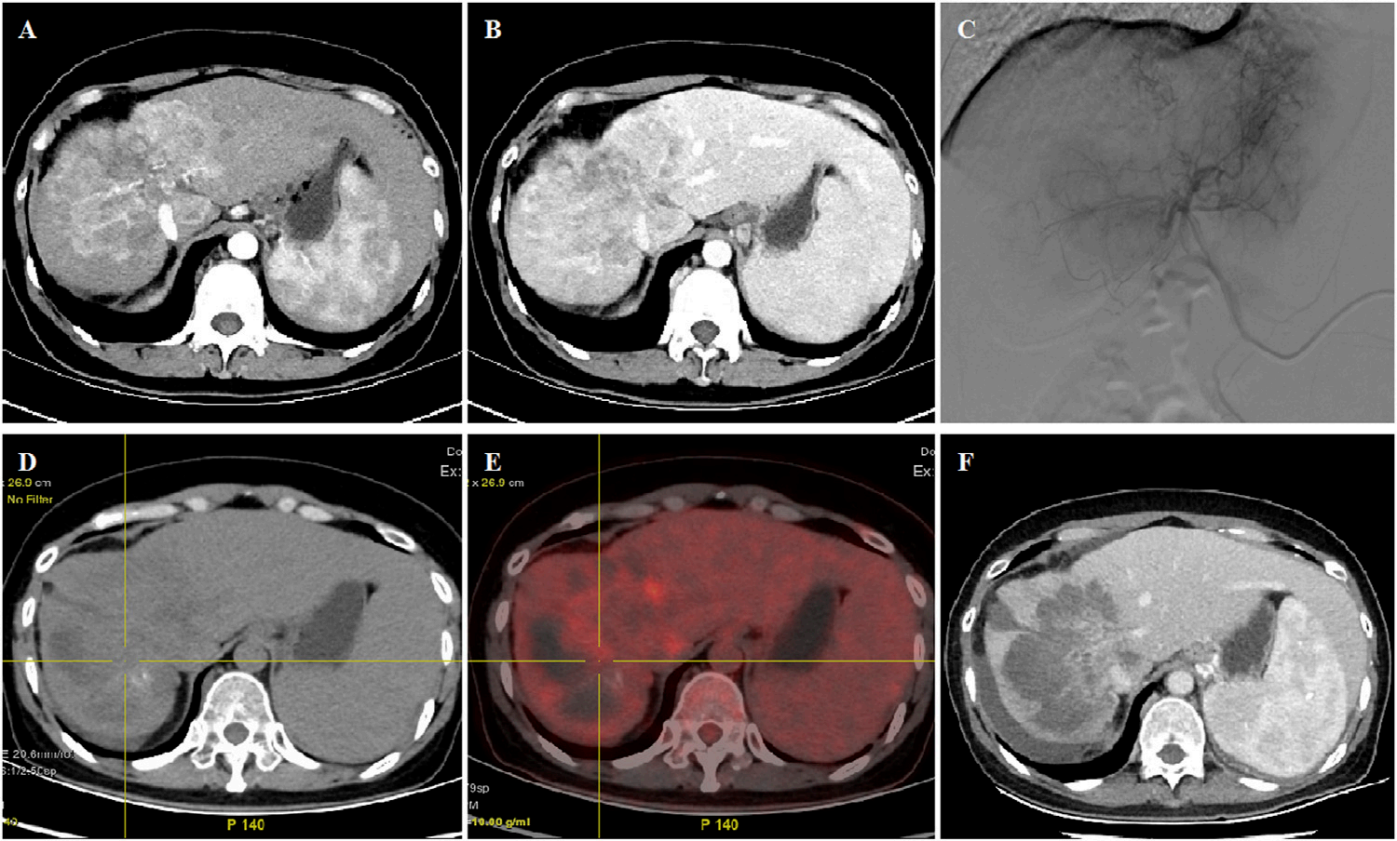
45 year old female patient treated with CalliSpheres beads for advanced primary HCC. **(A–B)** CT examination on admission revealed HCC in the right lobe. **(C)** Huge tumor staining was shown and was embolized by 300–500 μm oxaliplatin-loaded beads. **(D–E)** PET-CT showed significant reduction of the tumor and no residual tumor at 2.2 months’ follow-up. **(F)** Complete response was shown by mRECIST criteria at 5.0 months’ follow-up and still alive 15.5 months later.

**FIGURE 2:**
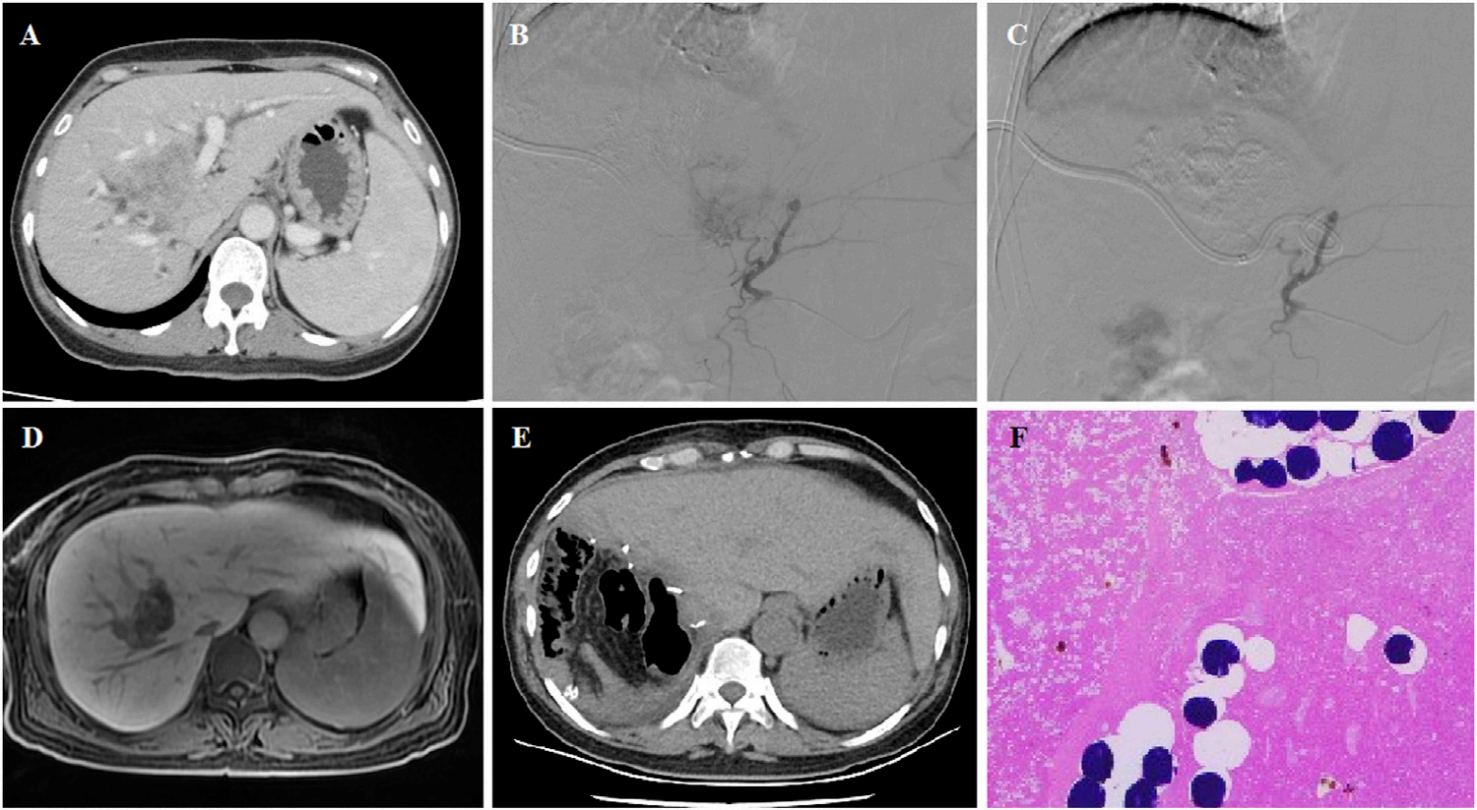
54 years female patient treated with CalliSpheres beads for advanced primary HCC. **(A)** CT revealed a tumor in the right lobe and the obstructed bile duct. **(B–C)** Tumor artery was super selectively incubated and embolized by 100–300 μm oxaliplatin-loaded beads. **(D)** MR revealed a shrunk tumor 4.6 months after DEB-TACE. **(E–F)** Tumor was resected 4.9 months after DEB-TACE, and no residual tumor was shown by pathological examination.

**FIGURE 3:**
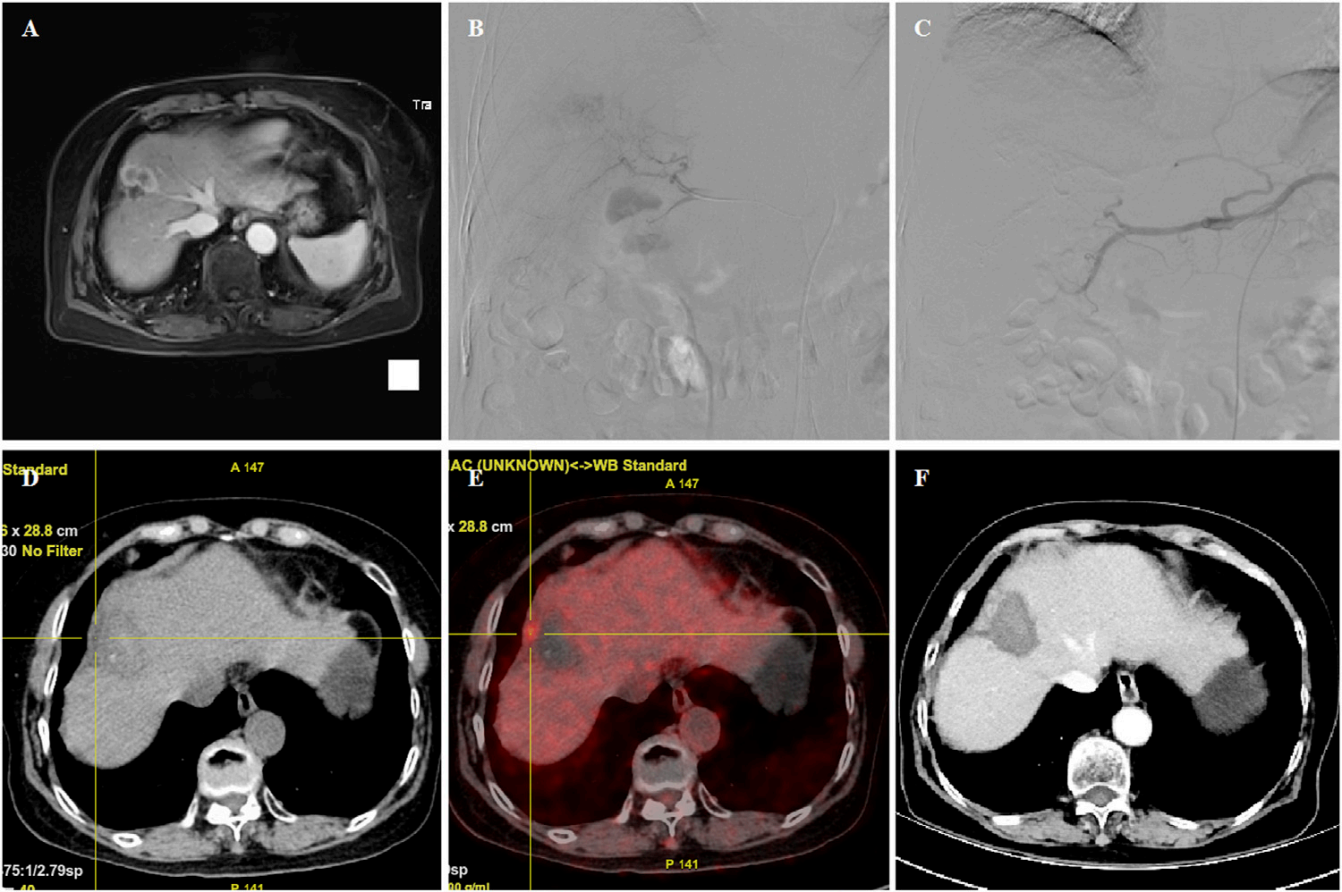
70 years female patient treated with CalliSpheres beads for advanced primary HCC. **(A)** MR revealed HCC in the right lobe. **(B–C)** Tumor staining was shown, and the right hepatic artery was embolized by 100–300 μm oxaliplatin-loaded beads. **(D–E)** PET-CT revealed a shrunk tumor with a small residual node 4.8 months later. **(F)** Tumor was found to shrink with no enhancement after 6.5 month follow-up. The patient passed away with tumor progression after 18.8 months.

Patients who were clinically or histologically diagnosed with primary HCC, either matter unresectable or recurrent tumor, were recruited from our department between November 2019 and November 2021. Contrast-enhanced computed tomography (CT) and/or magnetic resonance imaging (MRI) were carried out at baseline (Figures 1A,B; Figure 2A; Figure 3A). The inclusion criteria were 1) age 18–81 years, with a life expectancy more than 3 months; 2) Barcelona Clinic Liver Cancer (BCLC) stage B or C; and 3) Child–Pugh class A or B. Exclusion criteria were 1) allergy to study drugs; 2) liver metastatic cancer; 3) severe cardiovascular comorbidities including unstable angina, myocardial infarction, or uncontrolled hypertension; 4) coagulopathy or bleeding diathesis; 5) BCLC stage D; and 6) Child–Pugh class C. This study was approved by the Medical Ethics Committee of our hospital. Informed consent for the DEB-TACE procedure was obtained from all patients before the procedure.

### DEB-TACE Procedures

The femoral artery was accessed after local anesthesia and a 5F right hepatic catheter (Terumo, Japan) was introduced. Tumor-feeding vessels were catheterized and super-selected by a 2.7-F microcatheter (Progreat, Terumo, Japan). For diffuse lesions, the right hepatic and/or left hepatic artery was catheterized for embolization, avoiding the embolization of gallbladder artery and gastroduodenal artery. Oxaliplatin (100 mg) was pre-loaded with one vial of 100–300 μm or 300–500 μm CalliSpheres beads (Jiangsu Hengrui Medicine Co. Ltd., Jiangsu, China) for 30 min and then mixed with iodixanol at a ratio of 1:1. After infusion of raltitrexed (4 mg) or fluorouracil (500 mg), a bottle of CalliSpheres beads was used for embolization of the tumor-feeding vessels. Gelatin sponge particles or polyvinyl alcohol particles (Merit, American) were used if embolization was insufficient (Figure 1C; Figure 2BC; Figure 3BC).

### Efficacy and Safety Evaluation

The progression-free survival (PFS) was the primary endpoint. CT and/or MRI images were used for assessment of tumor response according to the response evaluation criteria in solid tumors (RECIST) (Eisenhauer et al., 2009) and modified RECIST (mRECIST) (Lencioni and Llovet, 2010). Patients were followed up by CT and/or MRI examination on 1, 3, and 6 months after the procedure and every 2 months thereafter until the end of the study, complete response, or patient death (Figures 1D–F; Figures 2D–F; Figures 3D–F). One patient was lost to follow-up in this study. The phone call follow-up was performed for the remaining 28 patients, and the last follow-up date was 20 January 2022. Disease control rate and safety were the secondary endpoints. Safety and toxicity were assessed according to the National Cancer Institute Common Toxicity Criteria (version 3.0) (Trotti et al., 2003).

## Results

### Patient Characteristics

Twenty-nine patients were enrolled in this study, including 20 males and nine females (mean age 58.1 ± 10.6 years). Disease characteristics and baseline demographics are shown in Table 1. Extrahepatic metastases were present in 13 patients (44.8%), and portal vein trunk or inferior vena cava invasion was found in five patients (17.2%). Eighteen patients (62.1%) complained of abdominal pain or distension, and median duration of symptom was 1.0 months. Twenty-one patients (72.4%) received synchronous treatments, including targeted therapy (*n* = 9), immunotherapy (*n* = 2), and targeted combined with immunotherapy (*n* = 10). For 10 patients who received combination treatment, targeted immunotherapy was used continuously.

**TABLE 1:** Patient characteristics at admission.

Parameters	Data
Male, n (%)	20 (69.0%)
Mean age, years	58.1 ± 10.6
Lesion types	
Right lobe	14 (48.3%)
Left lobe	1 (3.4%)
Bi-lobar	14 (48.3%)
Symptom duration, months	1.0 (0.4, 10.5)
Recurrence after surgery	5 (17.2%)
Systemic treatments	21 (72.4%)
Targeted therapy	9 (31.0%)
Immunotherapy	2 (6.9%)
Targeted and immunotherapy	10 (34.5%)
Hepatitis B virus infection	14 (48.3%)
BCLC stage B/C	8 (27.6%)/21 (72.4%)
Child–Pugh class A/B	20 (69.0%)/9 (31.0%)
Multinodular/bulky tumor/diffuse	5 (17.2%)/11 (37.9%)/13 (44.8%)
Portal vein trunk or IVC invasion	5 (17.2%)
Portal vein or hepatic vein branch invasion	7 (24.1%)
Extrahepatic sites	13 (44.8%)
Lung	2 (6.9%)
Lymph node	10 (34.5%)
Bone	2 (6.9%)
Spleen	1 (3.4%)
Tumor diameter, mm	91.6 ± 41.2
a-Fetoprotein level	24 (82.8%)
Normal	7 (24.1%)
<400 ng/ml	4 (13.8%)
400–10,000 ng/ml	8 (27.6%)
>10,000 ng/ml	5 (17.2%)

IVC = Inferior vena cava.

### DEB-TACE Treatments

As shown in Table 2, a total of 49 DEB-TACE sessions were performed in 29 patients, with a mean DEB-TACE cycle of 1.7 ± 0.9. Thirteen patients completed at least two cycles of DEB-TACE. All DEB-TACE procedures were successfully performed, with a technical success rate of 100%. Except for 13 sessions of DEB-TACE performed with CalliSpheres beads of 300–500 μm, the remaining 36 sessions of DEB-TACE were performed by using CalliSpheres beads of 100–300 μm in diameter. All patients received a bottle of beads; additional embolization was performed by 350–560 μm polyvinyl alcohol particles in four patients (13.8%) or 350–560 μm gelatin sponge particles in five patients (17.2%), or 300–500 μm embolization microsphere in six patients (20.7%). Thirteen patients (44.8%) received other interventional treatments, including conventional TACE (*n* = 8), percutaneous transhepatic cholangial drainage (*n* = 3), and thermal ablation in three patients (*n* = 3).

**TABLE 2:** Clinical data on DEB-TACE.

Variables	Data
Inpatient duration, months	9.0 (7.0, 12.0)
Hospitalization cost, ×10^4^ ¥	6.7 ± 2.0
DEB-TACE sessions	1.7 ± 0.9
Additional embolization	
Gelatin sponge particles	5 (17.2%)
Embolization microspheres	6 (20.7%)
Polyvinyl alcohol particles	4 (13.8%)
Complications, n (%)	21 (72.4%)
Fever	3 (10.3%)
Nausea	5 (17.2%)
Vomiting	3 (10.3%)
Thrombocytopenia	3 (10.3%)
hyperbilirubinemia	5 (17.2%)
Leukopenia	2 (6.9%)
Abdominal pain	12 (41.4%)
Raised ALT/AST	8 (27.6%)
Other interventional treatments, n (%)	13 (44.8%)
Conventional TACE	8 (31.0%)
PTCD	3 (10.3%)
^125^I seed implantation	1 (3.4%)
Thermal ablation	3 (10.3%)
Drainage of liver abscess	1 (3.4%)

ALT = Alanine aminotransferase; AST = Aspartate aminotransferase; TACE = Transcatheter arterial chemoembolization; PTCD = Percutaneous transhepatic cholangial drainage.

### Tumor Response

According to the RECIST criteria, only one patient achieved a complete response 3 and 6 months after DEB-TACE. Partial response was observed in one, four, and one patients at 1-, 3-, and 6-month follow-up. The overall response rates were 4.3, 26.3, and 15.4%, respectively, at 1, 3, and 6 months (Table 3). According to the mRECIST criteria, tumor enhancement decreased significantly after DEB-TACE, and higher rates of complete response and overall response were observed. The overall response rates were 52.4, 64.7, and 54.5% at 1, 3, and 6 months, respectively. The disease control rates were similar, whether assessed using RECIST criteria or mRECIST criteria (Table 4).

**TABLE 3:** Local tumor response assessed using RECIS criteria.

Response	1 month	3 months	6 months
Complete response	0 (0.0%)	1 (5.3%)	1 (7.7%)
Partial response	1 (4.3%)	4 (21.1%)	1 (7.7%)
Stable disease	21 (91.3%)	9 (47.4%)	5 (38.5%)
Progressive disease	1 (4.3%)	5 (26.3%)	6 (46.2%)
Overall response rate	1 (4.3%)	5 (26.3%)	2 (15.4%)
Disease control rate	22 (95.7%)	14 (73.7%)	7 (53.8%)

**TABLE 4:** Local tumor response assessed using mRECIS criteria.

Response	1 month	3 months	6 months
Complete response	4 (19.0%)	5 (29.4%)	3 (27.3%)
Partial response	7 (33.3%)	6 (35.3%)	3 (27.3%)
Stable disease	9 (42.9%)	2 (11.8%)	1 (9.1%)
Progressive disease	1 (4.8%)	4 (23.5%)	4 (36.4%)
Overall response rate	11 (52.4%)	11 (64.7%)	6 (54.5%)
Disease control rate	20 (95.2%)	13 (76.5%)	7 (63.3%)

### Survival

**FIGURE 4:**
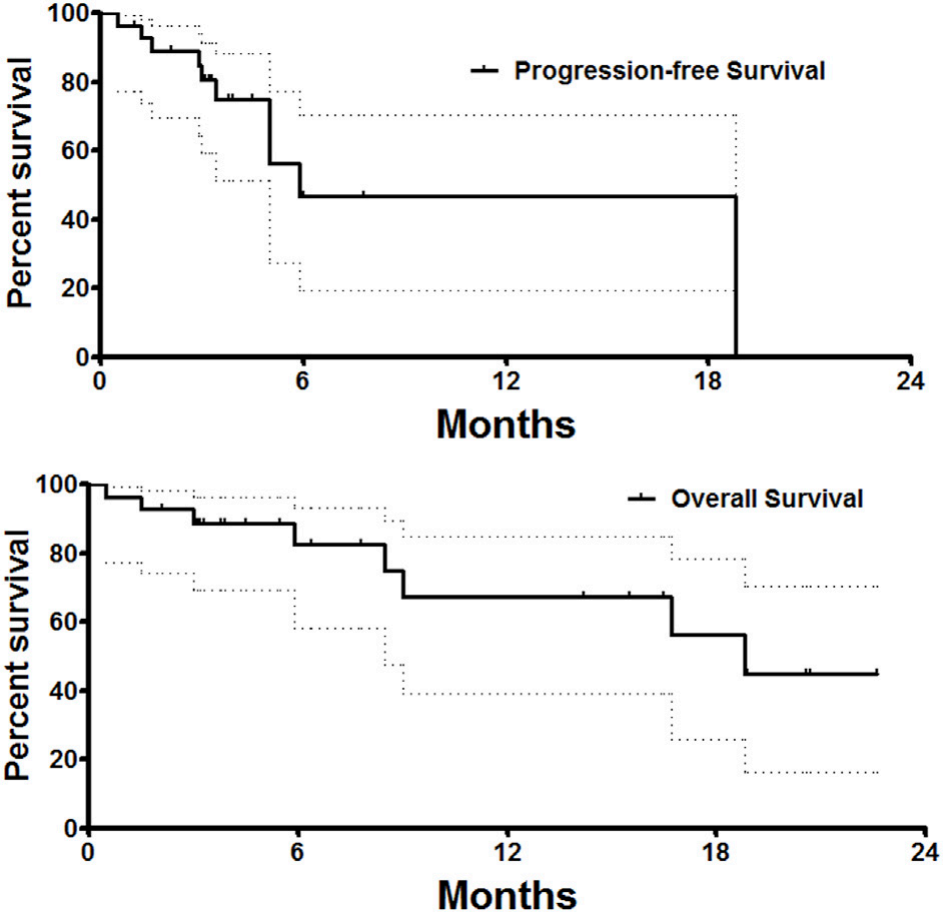
Survival follow-up. The PFS was 5.9 months, and the median overall survival was 18.8 months.

One patient was lost to follow-up, with a follow-up rate of 96.6%. The PFS was 5.9 months, and the median overall survival was 18.8 months (Figure 4). The 6- and 12-month overall survival rates were 82.5 and 67.5%, respectively. There was no significant difference in PFS between unresectable HCC and recurrent HCC (*p* = 0.41). The median overall survival was 18.8 months for unresectable HCC and 19.7 months for recurrent HCC, with no significant difference (*p* = 0.51). Twenty-one patients received DEB-TACE plus systemic therapy, and only nine patients received only DEB-TACE. The median overall survival was 18.8 months for patients who received only DEB-TACE and 16.7 months for patients who received DEB-TACE combined with targeted or immunotherapy. However, there was no significant difference in overall survival or PFS between these two groups.

### Safety and Toxicity

Minor complications were observed in 21 patients (72.4%), with no severe adverse events or treatment-related mortality. Abdominal pain (41.4%) was the most common treatment-related nonhematologic complication. All reported toxicities were grades 1 and 2.

## Discussion

HCC has the highest incidence in Southeast Asia and the major risk factor of HCC is hepatitis B virus transmission (McGlynn et al., 2015). More than 70% of HCC patients are diagnosed in the intermediate or advanced stage and cannot be treated with curative treatments such as liver transplantation or liver resection (Mazzaferro et al., 2014; Morise et al., 2014). For patients with BCLC tumor stage B, TACE is recommended as the first-line therapy (Bruix and Llovet, 2002). As the third generation of platinum anticancer drugs, oxaliplatin shows an excellent inhibitory effect on cancers (Ibrahim et al., 2004; Nobili et al., 2008). Oxaliplatin-based TACE, oxaliplatin combined with fluorouracil or doxorubicin, is usually used clinically for the treatment of HCC. However, adverse effects are observed due to high blood concentration, leading to cardiotoxicity and neurotoxicity (Bullinger et al., 2011) (Ransom et al., 2014).

As a relatively new drug-delivering device, DEB-TACE showed an advantage of sustained release of chemotherapeutic drugs, increased local drug concentration, and reduced toxicities (Bi et al., 2021b; Bi et al., 2021c). CalliSpheres microsphere is the first drug-eluting bead developed in China, which has been applied for loading and releasing oxaliplatin in an *in vitro* study (Han et al., 2019). Poggi et al. reported that DEB-TACE with oxaliplatin-eluting Hepasphere microspheres associated with chemotherapy is a safe and feasible treatment in advanced unresectable intrahepatic cholangiocarcinoma and liver metastasis of colorectal cancer (Poggi et al., 2008; Poggi et al., 2009). However, only nine patients with intrahepatic cholangiocarcinoma and eight patients with colorectal carcinoma liver metastases were enrolled, rather than those with primary HCC. Currently, no study reported the outcomes of oxaliplatin-eluting CalliSpheres microspheres in the treatment of HCC.

In this study, a total of 49 DEB-TACE sessions were performed in 29 patients with unresectable or recurrent HCC, with a technical success rate of 100%. The overall response rate and disease control rate were 52.4 and 95.2%, 64.7 and 76.5%, and 54.5 and 63.3%, respectively, at 1, 3, and 6 months after DEB-TACE according to mRECIST criteria. A higher overall response rate was determined by mRECIST criteria than by the RECIST criteria, which was consistent with our previous study (Zhao et al., 2016). The complete response was only one if assessed by RECIST, which was similar to the previous report (Rougier et al., 1997). The mRECIST may be more suitable for response assessment after DEB-TACE, considering that most patients showed a significant decrease in tumor enhancement after DEB-TACE procedure.

In this study, we also evaluated the safety of DEB-TACE loaded with oxaliplatin in unresectable or recurrent HCC patients. Our data suggested that abdominal pain (41.4%) was the most common treatment-related complication, which was similar to our previous study (Zhao et al., 2016). No treatment-related mortality or severe adverse events was observed in this study.

Doxorubicin is a regularly used drug in DEB-TACE. Huang et al. (Huang et al., 2021) reported the overall survival was 14.0 months for patients who received DEB-TACE with beads loaded with 60 mg doxorubicin, and the PFS was 6.3 months. These results were similar to the outcomes of our study. In addition, nausea/vomiting (18.8%), neutropenia (7.8%), and thrombocytopenia (12.5%) were observed in Huang’s study, which was also similar to our study.

There are some limitations in this study. Unresectable and recurrent HCC are different in prognosis and response to the treatment. However, the sample size of recurrent HCC was too small to compare the difference. Oxaliplatin release was very quick in CalliSpheres beads, and further studies are needed to prove the advantage of oxaliplatin-loaded DEB-TACE.

In conclusion, our preliminary results showed that DEB-TACE loaded with oxaliplatin-eluting CalliSpheres microspheres is safe and tolerable in patients with unresectable HCC.

## Data Availability

The raw data supporting the conclusions of this article will be made available by the authors, without undue reservation.
